# Punarnavayolepa Choornam in Iron Deficiency Anemia Management: Pharmaceutical Insights and Biological Activity

**DOI:** 10.7759/cureus.108160

**Published:** 2026-05-03

**Authors:** Jyothish G Nair, Rajani TS, Deepthy Mohan, Puthanpurakal Indu Chandrabose, Varsha Wilson, Atheena Biju Shyn, Remya Venugopala Menon, Ashi Augustine, Yadu Narayanan Mooss, Subrahmanya Kumar Kukkupuni, Sheela Karalam B, Chethala N Vishnuprasad, Asish Gopinathan Remanikutty

**Affiliations:** 1 Ayurveda Biology, Vaidyaratnam Ayurveda Research Institute, Thrissur, IND; 2 Ayurveda Biology and Holistic Nutrition, The University of Trans-Disciplinary Health Sciences and Technology, Bengaluru, IND; 3 Phytochemistry, Vaidyaratnam Ayurveda Research Institute, Thrissur, IND; 4 Botany, Christ College (Autonomous), Irinjalakuda, Thrissur, IND; 5 School of Humanities, National Institute of Advanced Studies (NIAS), Bengaluru, IND

**Keywords:** ayolepam, ayurveda pharmaceutics, boerhavia diffusa, drug design, iron bioavailability, iron deficiency anemia, punarnavayolepa choornam, traditional medicine

## Abstract

Background: Indigenous medical systems employ unique pharmaceutical techniques to meet the therapeutic needs. In the Indian System of Medicine, *Ayurveda*, a unique method called “*Ayolepam*” is described that facilitates efficient iron absorption from its source to the formulation. *Punarnavayolepa Choornam* (PC), prepared from *Boerhavia diffusa* L. (*Punarnava* in *Ayurveda*), is one such novel *Ayolepam* formulation developed by a traditional *Ayurveda* school of South India, widely used in the clinical management of iron deficiency anemia (IDA). This study aimed to scientifically evaluate the *Ayolepam* technique by assessing the iron-binding and bioavailability properties of PC, prepared from the leaves, root, and whole plant of *B. diffusa*, using in vitro model systems.

Methods: The iron content in both raw and processed samples of PC was quantified using inductively coupled plasma mass spectrometry (ICP-MS). A simulated in vitro digestion model was employed to assess the release of bioavailable iron from the formulation. Subsequently, iron bioavailability was evaluated using the human colorectal adenocarcinoma cell line (Caco-2) cell model of human intestinal epithelium following the ferrozine method.

Results: PC preparation from leaves, whole plant, and roots of *B. diffusa* showed a significant increase in the iron content compared to the raw material. In vitro digestion studies confirmed the efficient release of bioavailable iron from these formulations, and subsequent Caco-2 cell assays confirmed their iron bioavailability properties.

Conclusion: To conclude, the observations from our preliminary study provide a scientific rationale for this unique pharmaceutical preparation (PC) from a traditional school of Ayurveda, supporting its clinical application in IDA management.

## Introduction

Preparation of a formulation satisfying all pharmaceutical principles is extremely important and sometimes even challenging. Before the discovery of modern techniques and instruments, traditional medical practices across the globe established their own unique and innovative methods for preparing formulations to meet the pharmacodynamic and pharmacokinetic requirements. *Ayurveda*, an Indian System of Medicine (ISM), has described numerous methods to prepare drug formulations with optimal safety and efficacy [1]. One such method of drug preparation is called “*Ayolepam*” (in Sanskrit, ‘*Ayo*’ = iron and ‘*Lepam*’ = paste/ointment/smearing on a surface), wherein the raw materials (or ingredients) are ground into a paste and smeared over a cast-iron sheet and dried. The powder is scraped off and used for clinical administration [2,3]. While classical texts of *Ayurveda* pharmacology and pharmaceutics like *Rasa Tarangini *and *Rasa Ratna Samucchaya* mention three types of *Loha *(iron), *Teekshna Loha* (mild steel) is most frequently used in modern practice due to its easy availability [4].

Kerala, a state in the southern part of India, is known for its unique schools of *Ayurveda* practice with several unique formulations and procedures used for disease management [5]. One such unique school is called the “*Ashta Vaidya*” tradition, where the professionals trained in this school are known to have expertise in all eight branches of *Ayurveda* [6]. *Punarnavayolepa Choorna *(PC), the formulation of focus in this study, is one of the unique preparations from a renowned *Ashta Vaidya* school in Kerala; it is prepared following the pharmaceutics principles of *Ayolepam* technique [6]. PC is a single-herb-based preparation using the roots of *Boerhavia diffusa *Linn.(family Nyctaginaceae), referred to as *Punarnava* in *Ayurveda.* It is a well-known medicinal herb in ISMs and is also used in South America and Africa. Different parts of this plant, primarily the roots, are used for managing gynaecological diseases, hepatoprotection, and gastrointestinal disorders. In *Ayurveda, Boerhavia diffusa *Linn. is recognised as a *Rasayana*, and is found to be a key ingredient in 35 formulations [7].

The most common indication of PC is in the management of *Pandu Roga*, a disease described in *Ayurveda,* whose clinical manifestations are comparable to those of iron deficiency anemia (IDA). A notable pallor or yellowish-white colouring of the skin, signifying a decreased hemoglobin level and/or a drop in the number of red blood cells (RBCs), is characteristic of both Pandu Roga and IDA [8]. Globally, iron deficiency is attributed to 50% of anemia cases; it ranks 9th among the 26 risk factors in the Global Burden of Disease (GBD) 2000 project, contributing to over 841,000 deaths and about 35 million disability-adjusted life years (DALYs) lost [9]. The most recommended strategies for managing IDA are dietary changes and iron supplements [10]. Diet is the only source of iron for the human body, but the bioavailability of iron from diets is too low due to various reasons and impairments in iron bioavailability, leading to diseases like IDA; hence, iron-folic acid supplements are becoming imperative for health management. However, problems like poor absorption, gastrointestinal side effects, and low patient compliance frequently limit the use of oral iron supplements [11]. These challenges corroborate the need for better, safe, and culturally acceptable iron bioavailability enhancers for the masses. PC, a unique preparation of the *Ashta Vaidya* tradition of *Ayurveda*, is a choice in this direction, which is administered with buttermilk as an adjuvant. Although PC has been used in *Ayurveda* practice for several years for the clinical management of IDA-like disease conditions, there are no systematic studies available on its clinical efficacy and mode of action.

The primary objective of the present study is to address this gap by evaluating the pharmaceutical and pharmacological properties of PC with a focus on its ability to enhance iron bioavailability in Caco-2 cells, a well-known model for human intestinal absorption. Our research used an in-house-prepared PC derived from the roots, leaves, and the whole plant of *Boerhavia diffusa* Linn. Additionally, the formulation was analysed for its physicochemical properties and nutritional profile using appropriate experimental methods.

This article was previously posted to the bioRxiv preprint server on October 9, 2025 [12].

## Materials and methods

Plant material, cell line, and fine reagents

The *B. diffusa* plants were taxonomically identified by botanists at the Vaidyaratnam Ayurveda Research Institute, Thrissur, India, and the leaves, roots, and whole plants were collected separately from the raw material storage section of the Vaidyaratnam Oushadhasala, Thrissur. Caco-2 was a kind gift from Dr. Raghu Pullakhandam at the National Institute of Nutrition, Hyderabad. Minimum essential medium (MEM; cat. no. 11095072) and foetal bovine serum (FBS; cat. No. 10270106) were purchased from Thermo Fisher Scientific Inc. (Waltham, USA). All fine chemicals for various bioassays were purchased from Sigma-Aldrich (St. Louis, USA).

Mild-steel iron trays

The standard mild-steel iron sheets were procured from a commercial vendor (Shankara Build Pro, Thrissur, India), and the in-house maintenance team of Vaidyaratnam Oushadhasala manufactured the sheets into trays (with 40 × 40 × 6 cm, length × width × height).

*Punarnavayolepa Choornam* preparation 

The plant parts collected were washed thoroughly with clean water to remove any debris present. The iron trays were cleaned and washed with isopropyl alcohol before use to avoid contamination. The roots were dried and powdered, and made into a paste in a 1:1 ratio (powder:water, by weight). The leaves and the whole plant were made into a paste without drying in 1:1 ratio (plant part:water, by weight). All three samples (leaves, root, and whole plant) were smeared evenly on three separate iron trays and were dried in an oven at 40°C under controlled environmental conditions. The complete drying time for root, whole plant, and leaf *Ayolepam* preparations was 66, 113, and 88 hr, respectively. The powders were labelled and stored in air-tight glass containers for further studies.

Pharmacognosy studies

Both raw materials and *Ayolepa* preparations were subjected to pharmacognosy analysis. Macroscopic characteristics of plants were documented through visual examination of the leaf, stem, and root. Transverse section(s) (TS) of the leaf, stem, and root were prepared for histological investigations to examine anatomical structure. To find distinctive microscopic characteristics, powdered samples of the leaf, stem, and root were subjected to powder microscopy. Powder microscopy was also used on *Ayolepam* formulations made from the three parts of the plant.

Physicochemical analysis

Total ash and loss on drying (LOD) were calculated using the methods described in the Ayurvedic Pharmacopoeia of India [13]. The Anthrone technique, as outlined by Sadasivam and Manickam (2008), was used to measure total carbohydrates [14].

Micro- and macronutrient profiling of samples

For the nutrient profiling, 2 g of the samples were weighed and heated in a muffle furnace at 450°C for two and a half hours to make ashes of the sample. This ash was then treated with 5 mL of 2N HCl and slowly heated to ensure complete solubilization. The solution was left overnight undisturbed for complete reaction, and any insoluble residues were removed by filtration using filter paper. The filtered solution was transferred to a 50 mL volumetric flask and made up to 50 mL using distilled water. Standard solutions of calcium chloride, sodium chloride, and potassium chloride at linear concentrations of 25, 50, and 100 ppm were used to calibrate a flame photometer for analysis. We employed a µ Controller Based Flame Photometer 128 (Systronics India Ltd., Ahmedabad, India) as our instrument model, performed a linear calibration, and quantitatively evaluated the linearity and regression coefficient (R^2^) to determine the percentage of corresponding elements. The prepared sample solution was lastly examined to ascertain its mineral content, to provide an accurate and trustworthy nutritional evaluation.

Simulated in vitro digestion of samples

The in vitro digestion of PC was performed following the standardized protocol in the lab with minor modifications to suit the samples. The electrolyte solutions for simulated gastric fluid (SGF) and simulated intestinal fluid (SIF) were prepared as reported by Butala et al. [15]. The three varieties of PC formulations (prepared as mentioned in section *Punarnavayolepa Choornam* preparation) were taken (0.5 g of each preparation) in a 50 mL Falcon tube and suspended in 12.5 mL of SGF supplemented with an electrolyte solution containing KCl, KH_2_PO_4_, NaHCO_3_, NaCl, MgCl_2_·6H_2_O, and (NH_4_)_2_CO_3_, adjusted to pH 2.0 using 6N HCl, and supplemented with 2500 U/mL pepsin and 0.16 mM CaCl_2_·2H_2_O. Simulated intestinal fluid (SIF) contained an electrolyte solution composed of KCl, KH_2_PO_4_, NaHCO_3_, NaCl, and MgCl_2_·6H_2_O, adjusted to pH 7.0 using 5N NaOH, and supplemented with pancreatin (500 µg/mL), bile salts (3 mg/mL), and 0.6 mM CaCl_2_·2H_2_O incubated at 37°C in a shaking water bath for another two hours. After complete digestion, the samples were heat-inactivated by keeping them in a 65°C water bath for 30 minutes to stop all enzymatic activity. The digests were centrifuged at 5000 rpm for 15 min and filtered, and the supernatant was collected and stored at -80°C for further use.

Iron estimation using ICP-MS

Iron content was determined using inductively coupled plasma mass spectrometry (ICP-MS; ICP-MS MassHunter Workstation; Agilent Technologies, Santa Clara, USA). Samples (minimum 1.2 mL in the vial) were introduced via a concentric nebulizer with peristaltic uptake (~0.1-0.2 mL/min) under standard plasma conditions (RF power 1550-1600 W, sampling depth 8-10 mm, carrier gas 1.0-1.1 L/min, makeup gas ~0.1 L/min). Helium collision mode (He KED, 4-5 mL/min, 3-5 V) was used to remove ArO⁺ interference, with Fe monitored at m/z 56 (primary) and m/z 57 (confirmation). Rh-103 (or Sc-45/Ge-72) was employed as the internal standard. Dwell time was ~0.1 s with 30-50 sweeps and triplicate replicates per isotope. The calibration solution was prepared in 1% HNO₃ over an appropriate range (e.g., 1-100 µg/L) with internal standards added, and blanks, continuing calibration verification, and independent check standards were analyzed at regular intervals. The limit of detection for iron measurements was 0.05 ppm, and the limit of quantification was 0.165 ppm. The rinse solution was 1%-2% HNO_3 _between samples, with an extended rinse after high concentration runs. The internal standard and Helium collision mode (He KED) were utilized to ensure the precision and reproducibility of the iron measurements against spectral interferences. Results are reported directly from the ICP-MS MassHunter Workstation software with dilution factor corrections applied [16].

Evaluating the iron bioavailability using Caco-2 cells following the ferrozine method

Caco-2 cells were maintained in the MEM supplemented with 10% FBS and essential amino acids. For the assays, cells were seeded in 12-well plates and cultured for 21 days, with media changes every 48 hr, to allow proper differentiation required for in vitro intestinal absorption models [17]. On the day of assay, the cells were treated with 500 µL of the growth media containing 150 µg/mL of FeSO_4_ along with varying concentrations (30%, 20%, and 10% v/v) of the *Ayolepam* digests. Cells treated with 150 µg/mL of FeSO_4_ and 500 µM ascorbic acid were used as experimental control (positive), and cells treated with respective concentrations of *Ayolepam *digests without FeSO_4_ were used as a control to see the iron bioavailability directly from the sample. The FeSO_4_-ascorbic acid combination served as an experimental control for evaluating the ability of the monolayer to uptake iron from the source. The cells were incubated at 37°C for 18 hr. After the incubation, the media was discarded, and the cells were rinsed with 500 µL of ice-cold saline solution (140 mM NaCl, 0.9%) to remove residual media. After washing, 500 µL of the stop solution (140 mM NaCl containing 10 mM PIPES (piperazine-N,N′-bis(2-ethanesulfonic acid)) was added to each well and incubated for one to two minutes to stop the reaction. Following this, the cells were washed twice with 400 µL of the removal solution (stop solution + 5 mM bathophenanthroline disulfonic acid). After the final wash, 150 µL of the solubilization solution (0.5 N NaOH) was added to each well, and the cells were completely scraped and transferred into microcentrifuge tubes. For complete lysis and solubilization, the cells were incubated at 60°C in a water bath for 30 minutes. Total cell protein of the different samples was estimated using the Bradford assay.

For estimating the intracellular iron in different groups, 200 µL of the freshly prepared iron-releasing solution (a 1:1 mixture of 1.4 M HCl and 4.5% KMnO_4_) was added to the lysates, followed by incubation for two hours at 60°C in a water bath. After incubation, 100 µL of the iron detection reagent (6.5 mM ferrozine, 2.5 mM ammonium acetate, and 1 M ascorbic acid) was added to each tube, and the reaction mixtures were mixed thoroughly and centrifuged at 4000 rpm for five minutes. Then 100 µL of the supernatant was transferred to a 96-well plate, in duplicates, and the absorbance was measured at 550 nm using a UV-visible spectrophotometer to determine iron concentration. An iron standard curve was prepared from serially diluted 1 mg/mL FeSO_4_, and the total iron absorbed by the cells was represented as ‘µg of FeSO_4_ Eq iron/mg of protein’ [17]. All experiments were performed using three independent biological replicates (n = 3), with two technical replicates per condition in each experiment. The validity of this control was confirmed by the observed more than two-fold increase in iron absorption compared to the FeSO_4_ group, demonstrating the model's sensitivity to known bioavailability enhancers.

Statistical analysis

Statistical analysis was performed using one-way analysis of variance (ANOVA) to compare multiple treatment groups with the FeSO_4_ control. The assumption of homogeneity of variances was assessed using the Brown-Forsythe test before conducting ANOVA, and Dunnett’s multiple comparisons post hoc test was applied to evaluate differences between each treatment group and the control. All statistical analyses were carried out using GraphPad Prism (version 8.4.2; GraphPad, Boston, USA). A p-value of less than 0.05 was considered statistically significant.

## Results

Pharmacognosy studies

Morphology of B. diffusa

**Figure 1:**
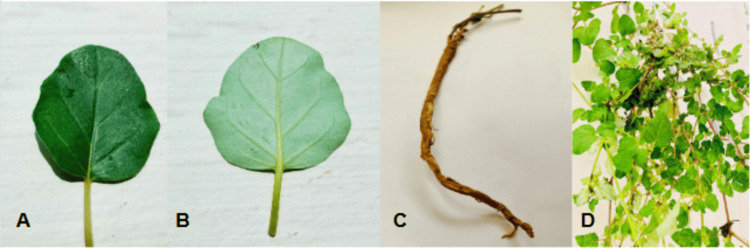
Morphological characteristics of the plant (A) Dorsal (adaxial) surface of a representative leaf, (B) ventral (abaxial) surface of the leaf, highlighting the detailed venation pattern, (C) the root system, illustrating its typical cylindrical structure, and (D) the overall habit of the plant, showing its characteristic branching pattern and foliage arrangement.

The leaves are grouped in opposing, unequal pairs, featuring a rounded or slightly pointed tip. The upper surface is green and glabrous, whereas the underside is pale, occasionally exhibiting a pinkish tint on the dorsal side. The leaf margins are entire or slightly undulate. The stem is slender, cylindrical, and stiff, exhibiting a greenish-purple coloration and swelling at the nodes, with a minutely pubescent texture. The root system is well-developed, fairly long, and somewhat tortuous, with a cylindrical shape measuring between 0.2 and 1.5 cm in diameter. The roots exhibit a yellowish-brown to brown coloration. The plant produces tiny, pink blooms that are either short-stemmed or almost sessile. The upper portion of the flower appears pink and funnel-shaped, and the inflorescence is made up of terminal and axillary panicles grouped in a corymb pattern. The fruit has a structure that is bluntly five-ribbed. These physical characteristics aid in the plant's identification and pharmacognostic assessment (Figure 1).

Transverse Section of the Leaf

**Figure 2:**
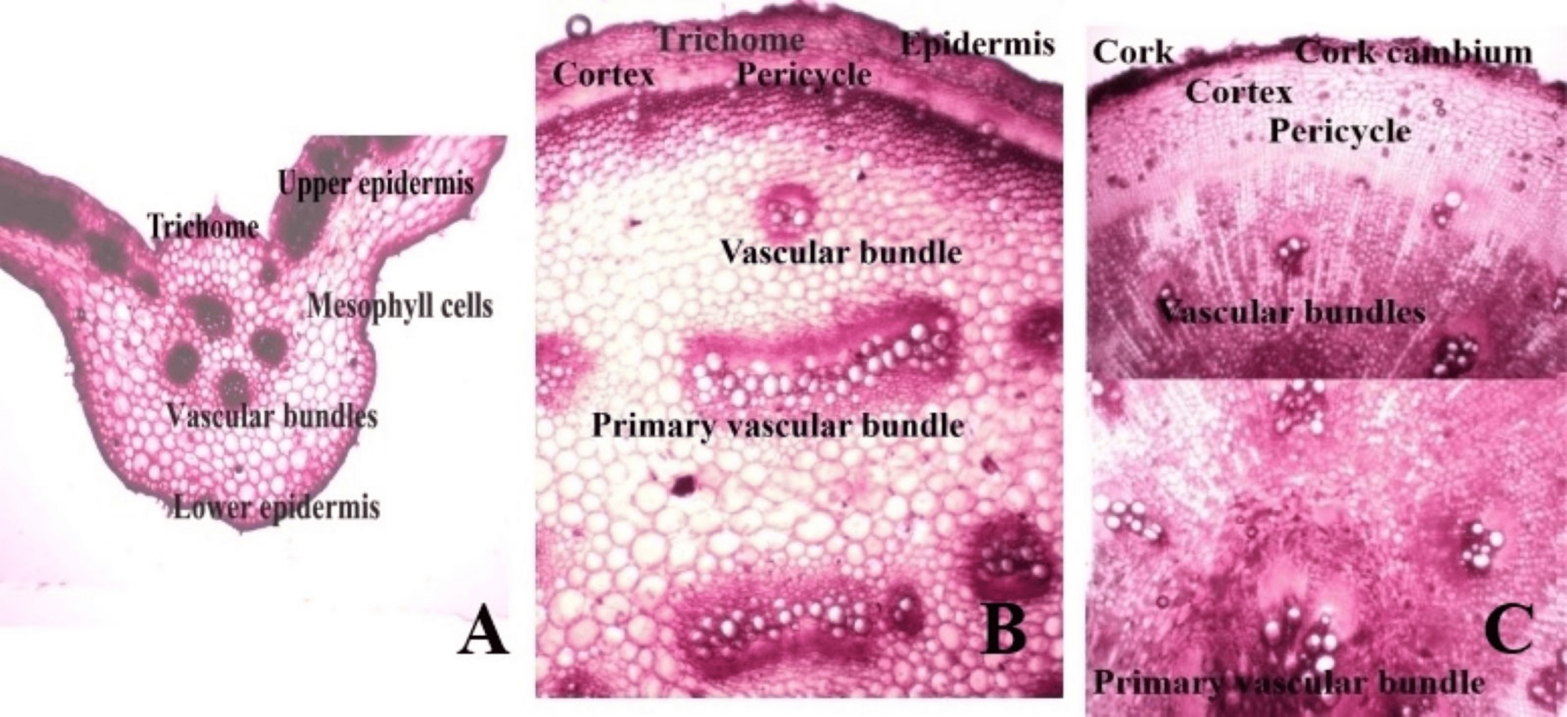
Anatomical features of Boerhavia diffusa in the transverse section (A) Leaf anatomy, (B) stem anatomy, and (C) root anatomy.

The TS of the leaf shows a dorsiventral structure. The outer walls of the epidermal cells show the position of crystalline granules of calcium oxalate beneath the thick cuticle. Numerous multicellular glandular trichomes are present on both surfaces of the leaf. Mesophyll is distinguished into one layer of palisade cells and two to four layers of loosely arranged parenchyma. Anomocytic stomata are present on both surfaces. The midrib of the leaf shows indistinct endodermis, one- to two-layered, thick-walled pericycle, often containing scattered, isolated fibres, stele consisting of many small vascular bundles often joined together in a ring, and many big vascular bundles scattered in the ground tissue, with intra-fascicular cambium present (Figure 2A).

Transverse Section of the Stem

The TS of the stem showed an epidermal layer bearing multicellular, uniseriate glandular trichomes. The cortex comprised one to two layers of parenchyma cells, with an indistinct endodermis. The pericycle was one- to two-layered, thick-walled, often containing scattered, isolated fibres. The stele exhibited numerous small vascular bundles arranged in a ring and many big vascular bundles scattered in the ground tissue. Intrafascicular cambium was present (Figure 2B).

Transverse Section of the Root

The TS of the mature root showed an outer cork layer composed of thin-walled tangentially elongated cells, with brown walls in the outer layers. The cork cambium consisted of one to two layers of thin-walled cells. The secondary cortex comprised two to three layers of parenchymatous cells followed by a cortex composed of 5-12 layers of thin-walled, oval to polygonal cells. Below the cortex, several concentric bands of xylem tissue alternated with a wide zone of parenchymatous tissue. The number of xylem bands varied according to the thickness of the root and was composed of vessels, tracheids, and fibres. Vessels were predominantly arranged in radial groups of two to eight, exhibiting simple pits and reticulate thickening. Tracheids were small, thick-walled, with simple pits. The central regions contained primary vascular bundles. Numerous calcium oxalate raphides, in single or in groups, were observed in the cortical region and parenchymatous tissue between xylem tissue. Starch grains, both simple and compound, having two to four components, were abundant throughout the cortical cells (Figure 2C).

Simulated in vitro digestion of PC samples and iron release

The samples' iron content was measured via ICP-MS to evaluate the differences in iron availability among various samples. The samples included blank (untreated), raw materials (leaf, whole plant, and root), water extract of PC, and in vitro digested PC. 

The iron concentration of raw materials varied across plant parts, with the root showing the highest content (1458.77 ppm), followed by the whole plant (1021.68 ppm) and leaves (290.57 ppm). Remarkably, after undergoing the *Ayolepam* process, all three preparations exhibited a several-fold increase in iron content, with leaves showing the highest (10504.79 ppm) content, followed by the whole plant (7353.95 ppm) and roots (5568.22 ppm). The findings are summarized in Table 1.

**Table 1:** Iron concentration in Boerhavia diffusa materials and formulations Values are expressed as means ± SDs (n = 5). Relative SD is less than 1. The table presents the iron concentration, measured in parts per million (ppm), across various samples. These include raw plant materials (*Boerhavia diffusa* leaf, whole plant, and root), *Punarnavayolepa* powder preparations, their corresponding water extracts, and extracts subjected to simulated in vitro digestion. A key finding is that the simulated digestion of *Punarnavayolepa Choornam* (PC) samples resulted in significantly higher levels of released iron compared to the untreated water extracts. Blank values were included as controls for the analysis.

Process	Sample	Iron concentration (ppm)
Blank	Blank	26.78 ± 0.24
Raw materials	Leaves	290.57 ± 1.81
	Whole plant	1021.68 ± 5.43
	Root	1458.77 ± 8.18
*Punarnavayolepa* powder	Leaves	10504.79 ± 95.2
	Whole plant	7353.95 ± 43.08
	Root	5568.22 ± 20.36
Water extract of PC	Leaves	107.58 ± 0.4
	Whole plant	47.33 ± 0.3
	Root	31.81 ± 0.28
Digested samples of PC	Leaves	115.52 ± 0.42
	Whole plant	124.28 ± 0.48
	Root	96.88 ± 0.39

In order to understand the iron release from the PC, the samples were subjected to a simulated in vitro digestion, and the digests were subjected to ICP-MS analysis. Alongside, a water extract of each sample, with the same amount, was prepared to compare the effect of iron release upon digestion. The iron concentration in the digested samples, regardless of the parts of *B. diffusa*, showed a statistically significant increase in iron compared to the water extract. The digests of whole plants showed 124.28 ppm, while the leaves and roots showed 115.52 and 96.88 ppm, respectively. At the same time, water extracts of the whole plant, leaves, and roots showed 47.33, 107.58, and 31.81 ppm of iron release, respectively (Table 1).

Improved bioavailability of iron in Caco-2 cells with PC digests

**Figure 3:**
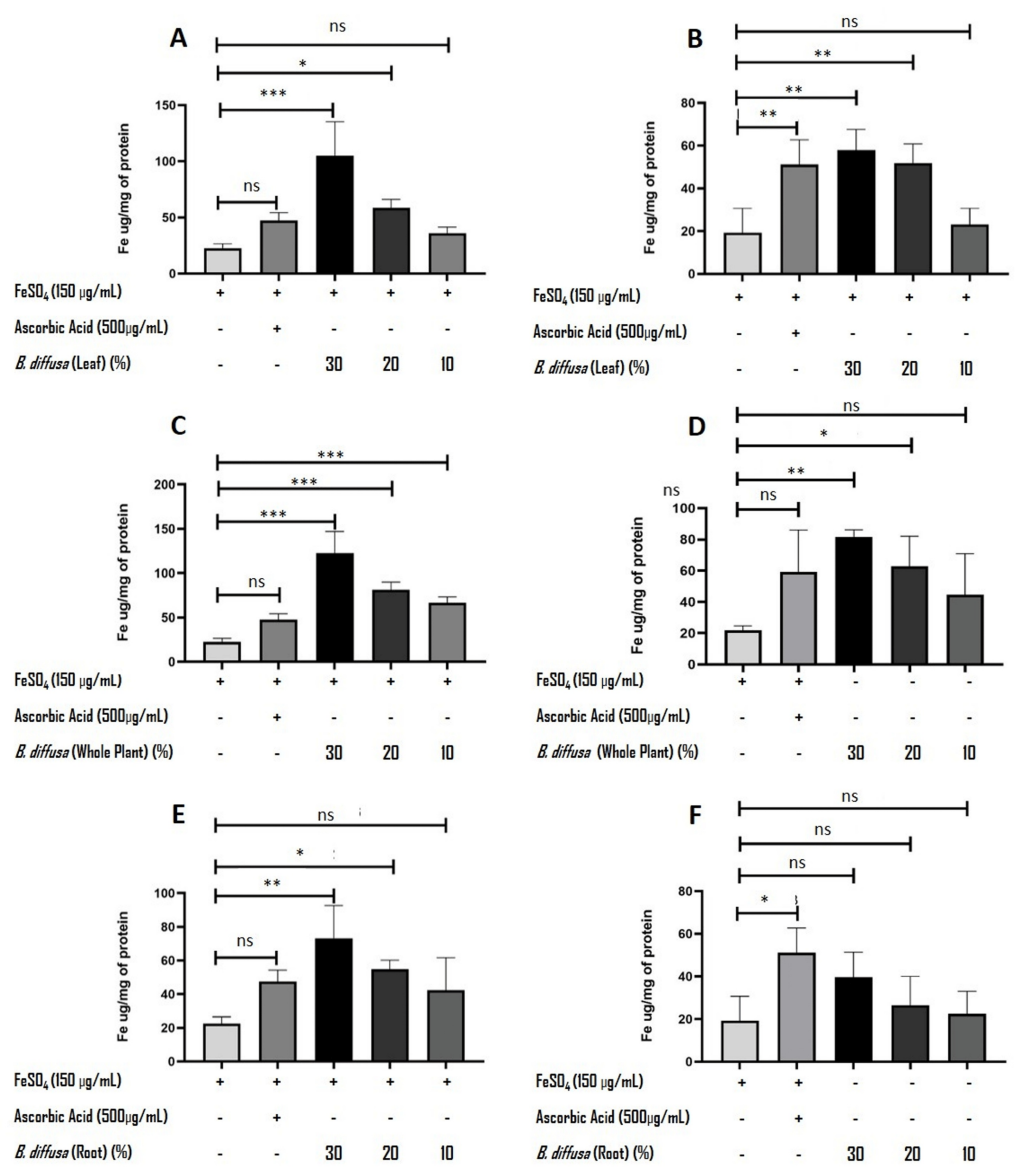
Effect of Boerhavia diffusa extracts on the cellular iron uptake in Caco-2 cells: intracellular iron concentration (µg/mg protein) in Caco-2 cells following treatment with Punarnavayolepa Choorna (PC) digests of B. diffusa leaf (A, B), whole plant (C, D), and root (E, F), in the presence (A, C, E) and absence (B, D, F) of FeSO₄ (150 µg/mL) Ascorbic acid (500 µg/mL) was used as a positive control. Data are presented as means ± SDs (n = 3). Statistical analysis was performed using one-way ANOVA followed by Dunnett’s multiple comparisons test, comparing all treatment groups to the FeSO_4_ control. Statistical significance is indicated as follows: *p < 0.05, **p < 0.01, ***p < 0.001, ns = not significant.

Treatment of Caco-2 cells with PC digests, with or without FeSO_4_, showed a concentration-dependent increase in intracellular iron concentration as quantified by the ferrozine assay. Cells treated with 150 μg/mL of FeSO_4_ showed a mean intracellular iron concentration of 22.59 ± 3.99 μg/mg of protein in one group (Figures 3A, 3C, 3E) and 19.27 ± 11.41 μg/mg of protein in the second group (Figures 3B, 3D, 3F). Treatment with all three samples of PC digests showed an increase in intracellular iron when compared to the FeSO_4_ control. Among the preparations, the whole-plant extract demonstrated the highest intracellular iron concentration at 30% (122.72 ± 24.32 μg/mg of protein), followed by the leaf-based *Ayolepam* extract (104.98 ± 30.29 μg/mg of protein) and the root extract (73.18 ± 19.50 μg/mg of protein). Cells treated with samples alone (without added FeSO_4_) showed a similar trend, but with a much lower amount of intracellular iron: 57.95±9.70 μg/mg of protein, 81.67±4.54 μg/mg of protein, and 39.68±11.68 μg/mg of protein, respectively, for *B. diffusa* leaves, whole plant, and roots. The lower concentrations of the sample showed a proportionate reduction of the intracellular iron concentration, showing a linear relationship. One-way ANOVA revealed statistically significant differences among treatment groups across all datasets (p < 0.05), indicating that the treatments significantly influenced the measured response. The F-values ranged from 3.81 to 27.2, with corresponding R² values between 0.604 and 0.916. While all the concentrations of PC tested showed an evident increase in iron bioavailability, Dunnett’s multiple comparisons test demonstrated that higher concentrations (30% and 20%) of the leaf and whole plant showed statistically significant differences compared to the control. Lower concentrations (10%) of all test samples were generally not significant. In contrast, roots exhibited limited or non-significant differences across the tested concentrations. Furthermore, co-treatment with FeSO_4_ was found to enhance the effects, particularly in leaf and whole-plant samples, where statistically significant differences were more pronounced (p < 0.05 to p < 0.001). The results clearly indicate the iron bioavailability-enhancing property of the preparation. Cells treated with 500 μg/mL of ascorbic acid, used as a positive control, showed a clear increase (more than two-fold) in iron absorption compared to the FeSO_4_ control, but the post hoc analysis showed non-significance in some of the test groups.

The results of powder microscopy, physicochemical analysis, and nutritional profiling are presented in the Appendices.

## Discussion

IDA is one of the major public health problems in developing countries. India, the country with the world's largest adolescent population, has 67.1% of children and 59.1% of adolescent girls suffering from anemia according to the 5th National Family Health Survey. The global scenario is not very different, and statistics show that around 1.62 billion people are affected by anemia [18]. While there are several strategies adopted across the globe for tackling IDA, the net result is not very encouraging, as evidenced by the statistics. The major drawbacks associated with the current management strategies are poor bioavailability and assimilation of iron from diet and therapeutic interventions, as well as low patient compliance with oral iron supplements [19]. To overcome these challenges, innovative pharmaceutical strategies can be adopted to develop interventions/supplements with better bioavailability, bioassimilation, and patient compliance. In this direction, our study presents a unique pharmaceutical preparation, *Punarnavayolepa Choornam*, and its potential applications in IDA management.

The uniqueness of the PC is its pharmaceutics. It is a preparation of the “*Ashta Vaidya*” school of *Ayurveda* from South India, where a single plant (*Boerhavia diffusa* - *Punarnava*) is processed on iron trays to entrap iron into the formulation. While the classical preparation uses the roots of *B. diffusa*, the current study explores the leaf and whole plant as well. The pharmaceutical process showed a marked increase in iron concentration in PC prepared from leaf, whole-plant, and root samples following the *Ayolepam* process. This increase in the amount of iron in the PC preparation indicates the intrinsic iron-binding potential of the *B. diffusa* plant. It is quite interesting to note that in the raw state, higher amounts of iron were found in the root, followed by the whole plant and leaves, whereas PC preparation following the *Ayolepam* procedure showed the reverse order, with leaf preparation having the highest amount of iron, followed by the whole plant and roots. More studies need to be carried out to understand the chemistry underlying this phenomenon of differential iron-binding ability of different parts of a particular plant. Nevertheless, the fact that the root contains higher amounts of iron probably justifies the rationale of selecting *B. diffusa* roots for PC preparation in the *Ashta Vaidya* school of pharmaceutics.

One of the major challenges in IDA management is the presence of anti-nutritive factors in dietary ingredients that reduce the iron bioavailability. These metabolites are known to sequester iron during gastrointestinal digestion and significantly reduce the release of iron for absorption by intestinal cells [20]. To understand the dynamics of PC during digestion, a simulated in vitro digestion model was employed in this study. The prepared PC (with leaf, root, and whole plant) was subjected to simulated in vitro digestion, and the amount of iron released into the soluble part of the digest was measured. This was compared with a sham digestion with an equivalent amount of distilled water. This step is critical, as plant-derived iron is typically complexed with phytates, fibres, proteins, and polyphenols, rendering it insoluble and poorly available for absorption [21]. Here again, the observation was quite interesting; the leaf-based PC preparation showed a similar amount of iron release with water and complete digestion, whereas both the whole plant and the root showed a significant increase in iron release after digestion, compared to the sham (water extract). This result suggests that *B. diffusa* is a plant that not only increases the absorption of iron from the source (here, for PC preparation, the iron tray), but also releases iron after digestion and makes it ready for bioavailability. This makes PC an ideal pharmaceutical preparation for IDA management.

The classical preparation of PC uses *B. diffusa* roots, but our study observed that the iron content is higher in leaf and whole-plant-based preparations. It is intuitive to assume that replacing *B. diffusa* roots with its leaf or whole plant may impart better pharmacological properties to PC with respect to IDA management. However, it is important to note the possible anti-nutritive factors (ANFs) present in different parts of *B. diffusa* that may hamper the bioavailability and assimilation of iron. The aerial part of *B. diffusa* contains tannins, phenols, and phytic acid that have been shown to inhibit iron absorption, especially non-heme iron, rendering it unavailable for assimilation [22]. Studies indicate that compared to leaves and stems, *B. diffusa* roots exhibit the lowest concentration of these ANFs with tannins (0.45%), saponins (0.77%), and phenols (0.09%) [23]. Similarly, *B. diffusa* leaves also contain high levels of minerals, particularly calcium (667 mg/100 g). Such elevated calcium levels are also shown to potentially compete with iron and inhibit its absorption [24].

Additionally, it is very important to note that *B. diffusa* roots contain some of the beneficial phytoconstituents, such as rotenoids, flavonoids, flavonoid glycosides, xanthones, purine nucleosides, lignans, ecdysteroids, and steroids [25]. Many of these compounds have been studied in facilitating non-heme iron absorption and mobilization through antioxidant activity, chelation, or interaction with cellular transport mechanisms. Studies indicate that the root of *B. diffusa* has the maximum antioxidant activity compared to the leaf and stem samples [26]. Our iron bioavailability study using Caco-2 cells demonstrates that treatment with digests from all three samples of the PC preparation effectively enhanced intracellular iron uptake compared to the FeSO_4_ control, suggesting a superior bioavailability. While this enhanced uptake is beneficial, it is crucial to maintain moderate iron supplementation. It is also a known fact that an excess amount of iron in the body impacts the metabolic homeostasis negatively by damaging various organs (hemochromatosis), and therefore, it is important to have moderate iron supplementation where the body absorbs maximum and reduces the wastage [27]. Although we do not have any clear justification for the *Ayurvedic *perspective of opting for *B. diffusa* roots over leaf or whole plant, ignoring the possible sustainable harvesting challenges, our analysis provides an acceptable explanation for the use of *B. diffusa* roots for PC preparation. The root-based preparations are clinically proven, and the mode of action studies also support the clinical claims. However, more detailed studies can probably shed more light on the efficacy and suitability of leaf- or whole-plant-based PC compared to root, from both pharmacology and pharmaceutics points of view.

The Recommended Dietary Allowance (RDA) for iron set by the US National Institutes of Health is set at 8 mg/day for healthy adult men and 18 mg/day for premenopausal women [28]. The ICMR-National Institute of Nutrition (NIN) recommends 19 mg/day iron for adult men and 29 mg/day for non-pregnant, non-lactating women, which is higher than the international standards considering the lower bioavailability of non-heme iron in the typical Indian diet [29]. Our PC preparations can provide support in meeting the RDA for iron for populations at risk of IDA. PC root preparation containing 5.57 mg of iron per gram, administered at a daily dose of 10 g, provides an adequate amount to meet the requirements. Also, for treating IDA, Ashtavaidya's practice of giving buttermilk as the adjuvant for PC again supports improving the bioavailability of iron. The fermented buttermilk can reduce phytates and other ANFs, which otherwise can limit iron absorption and can enhance the micronutrient availability, including iron and zinc [30]. The milk fat globule membrane, with components such as sphingolipids and gangliosides that modulate iron transport, accompanied by antioxidant and reducing activity from high sulfhydryl content, binds and retains iron in the absorbable ferrous (Fe²⁺) state. Furthermore, the presence of phosphoserine residues in milk proteins (especially caseins) helps in strong iron binding: fermentation again hydrolyzes these proteins, while the antioxidants protect iron from oxidation and lipid peroxidation, to boost intestinal iron uptake [31].

While the study successfully answers the research objectives, integrating traditional *Ayurvedic* pharmaceutics with modern analytical models like Caco-2 has certain limitations. The absence of a sham iron-tray control hinders the ability to fully identify the specific role of the plant material in the iron-trapping process. Furthermore, additional confirmatory experiments, including clinical trials, are essential to understand the systems-level effect of the formulation in enhancing the iron bioavailability. However, since PC is a formulation widely used in *Ayurveda *clinical management, our research acts as a reverse pharmacology approach to provide molecular level validation of the benefits observed in clinical practice.

## Conclusions

In conclusion, our results present a novel pharmaceutical product design from a traditional school of *Ayurveda *and provide preliminary scientific evidence that strongly supports the effectiveness of the traditional *Ayolepam* preparation in improving iron incorporation into the formulation, release upon digestion, and bioavailability, using *Punarnavayolepa Choorna* as an example.

Our study reaffirms the traditional use of *Punarnava* as a *Rasayana* herb in Ayurveda and highlights its potential in IDA management. A comparative evaluation of leaf, whole-plant, and root preparations indicates that while iron content varies, bioavailability depends on the phytochemical makeup and processing. The root-based formulation shows a balanced and consistent profile that supports its traditional use. The ability of PC to improve iron uptake highlights its potential as a helpful addition to regular iron supplementation. Given the global burden of iron deficiency anemia, further translational studies, including in vivo and clinical trials, are required to substantiate its efficacy and safety as a functional nutraceutical intervention for IDA.

## Appendices

Appendix 1: Powder microscopy

**Table 2:** Comparative powder microscopic characteristics of raw leaf and processed leaf (Ayolepa leaf) samples

No.	Microscopic features	Raw leaf	Processed leaf
1.	Starch grains	Present	Present
2.	Fragment of mesophyll cells with contents	Present	Present
3.	Prismatic crystal	Present	Present
4.	Acicular crystals	Present	Comparatively lesser
5.	Oil globule	Present	Present
6.	Trichome	Present	Present
7.	Dark-coloured cellular masses		Numerous
8.	Stomata	Present	Present

**Table 3:** Comparative powder microscopic characteristics of raw root and processed root (Ayolepa root) samples

No.	Microscopic features	Raw root	Processed root
1.	Starch grains	Numerous	Numerous
2.	Acicular crystals	Numerous	Comparatively lesser
3.	Prismatic crystals	Present	Present
4.	Vessel fragments	Present	Present
5.	Fibres	Present	Present
6.	Tracheids	Present	Present
7.	Oil globule	Present	Present

**Table 4:** Comparative powder microscopic characteristics of raw whole-plant and processed whole-plant (Ayolepa whole plant) samples

No.	Microscopic features	Raw whole plant	Processed whole plant
1.	Starch grains	Numerous	Present
2.	Acicular crystals	Numerous	Numerous
3.	Prismatic crystals	Present	Present
4.	Vessel fragments	Present	Present
5.	Fibres	Present	Present
6.	Tracheids	Present	Present
7.	Oil globule	Present	Present
8.	Fragment of mesophyll cells with cellular contents	Present	Present
9.	Trichome	Present	Present

Appendix 2: Physicochemical analysis

The physicochemical examination of the formulation was conducted to determine its moisture content, total ash value, and carbohydrate composition, with the results provided in Table 5.

**Table 5:** Moisture content, total ash value, and carbohydrate composition of different samples LOD: loss on drying

No.	Sample details	LOD %	Total ash %	Total carbohydrate %
1.	Raw leaves	4.61%	17.53%	77.86%
2.	Leaves (paste smeared)	2.62%	18.67%	78.71%
3.	Raw whole plant	3.65%	9.65%	86.70%
4.	Whole plant (paste smeared)	1.85%	12.21%	85.94%
5.	Raw root	3.32%	6.92%	89.76%
6.	Root (paste smeared)	2.57%	8.70%	88.73%

Appendix 3: Nutritional profiling

**Table 6:** Nutrition profile of different samples based on the different parts of the Punarnava plant Sodium, potassium, and calcium levels are calculated in ppm.

No.	Sample details	Sodium	Potassium	Calcium	Calorific value
1.	Raw leaves	0.36 ppm	5.44 ppm	1.80 ppm	311.44
2.	Leaves (paste smeared)	0.31 ppm	4.51 ppm	1.81 ppm	314.84
3.	Raw whole plant	0.35 ppm	2.78 ppm	1.17 ppm	346.8
4.	Whole plant (paste smeared)	0.33 ppm	2.74 ppm	1.66 ppm	343.76
5.	Raw root	0.38 ppm	0.98 ppm	1.40 ppm	359.04
6.	Root (paste smeared)	0.33 ppm	1.14 ppm	1.77 ppm	354.92
